# A Current Review of the Meniscus Imaging: Proposition of a Useful Tool for Its Radiologic Analysis

**DOI:** 10.1155/2016/8329296

**Published:** 2016-02-11

**Authors:** Nicolas Lefevre, Jean Francois Naouri, Serge Herman, Antoine Gerometta, Shahnaz Klouche, Yoann Bohu

**Affiliations:** ^1^Clinique du Sport Paris V, 75005 Paris, France; ^2^Institut de l'Appareil Locomoteur Nollet, 75017 Paris, France

## Abstract

The main objective of this review was to present a synthesis of the current literature in order to provide a useful tool to clinician in radiologic analysis of the meniscus. All anatomical descriptions were clearly illustrated by MRI, arthroscopy, and/or drawings. The value of standard radiography is extremely limited for the assessment of meniscal injuries but may be indicated to obtain a differential diagnosis such as osteoarthritis. Ultrasound is rarely used as a diagnostic tool for meniscal pathologies and its accuracy is operator-dependent. CT arthrography with multiplanar reconstructions can detect meniscus tears that are not visible on MRI. This technique is also useful in case of MRI contraindications, in postoperative assessment of meniscal sutures and the condition of cartilage covering the articular surfaces. MRI is the most accurate and less invasive method for diagnosing meniscal lesions. MRI allows confirming and characterizing the meniscal lesion, the type, the extension, its association with a cyst, the meniscal extrusion, and assessing cartilage and subchondral bone. New 3D-MRI in three dimensions with isotropic resolution allows the creation of multiplanar reformatted images to obtain from an acquisition in one sectional plane reconstructions in other spatial planes. 3D MRI should further improve the diagnosis of meniscal tears.

## 1. Introduction

Arthroscopic knee surgery is the gold standard in the diagnosis and treatment of intra-articular knee lesions. Preoperative imaging is still necessary before any surgery. Indeed, the diagnostic arthroscopy alone has no place in the evaluation of meniscal lesions of the knee. Clinicians (sports doctor, surgeon, or rheumatologist) therefore need to have a precise radiological analysis of meniscal lesions and associated injuries in order to best adapt their treatment. In the literature, many diagnostic radiological examinations were described for the evaluation of meniscal lesions but the magnetic resonance imaging (MRI) is the most accurate and least invasive for the diagnosis of meniscal tears. This technique has revolutionized the imaging of the knee and has become the “gold standard” imaging of the meniscus. Studies have shown excellent results regarding the sensitivity and specificity of MRI in the diagnosis of meniscal tears. They are used to classify the different meniscal lesions, particularly in the early detection of grade I and grade II lesions to reduce the rate of unnecessary diagnostic knee arthroscopy. This paper describes the various complementary tests used today in the diagnosis of meniscal tears with a precise description of all lesions. All anatomical descriptions were clearly illustrated by MRI, arthroscopy, and/or drawings.

## 2. Standard Radiography

The value of standard radiography is extremely limited for the assessment of meniscal injuries because the meniscus is not normally visualized with this type of examination. Standard radiography is therefore not useful in the investigation and diagnosis of meniscal injuries. Nevertheless, conventional X-ray of the knee may be indicated to confirm or obtain a differential diagnosis such as osteoarthritis, which often develops in association with meniscal degeneration. AP and lateral X-rays should be performed in the unipodal stance, as well as a standing flexion view (Schuss view) to evaluate and compare the height of the joint space of the weight-bearing area compared to the contralateral side. Thus, a radiographic examination is recommended in case of suspected meniscal injury in patients over the age of 50 because of the risk of associated osteoarthritis. Joint space narrowing of more than 50% or even complete narrowing can create doubt about a potential clinically symptomatic meniscal injury. Radiography can also exclude unsuspected lesions such as osteochondritis or loose bodies. Finally, in the presence of a discoid meniscus, X-rays can identify the relative widening of the involved joint compartment, usually the lateral compartment. Radiography can be used to assess the quality of the bone stock, the width of the tibiofemoral joint spaces, and the thickening of the medial or lateral tibial plateau.

## 3. Ultrasound

Ultrasound of the knee is a highly valuable diagnostic tool for tendon (patellar tendon, quadricipital tendon, and pes anserinus) and peripheral ligament injuries (medial and lateral collateral ligaments) [1]. Visualization of joint effusion (hydrarthrosis or hemarthrosis) and cysts (which do or do not communicate with the joint) is also good on ultrasound. On the other hand, ultrasound is rarely used as a diagnostic tool for meniscal pathologies. De Flaviis et al. [2] reported a sensitivity of 82% with dynamic ultrasound for the detection of meniscal degeneration based on criteria including meniscal irregularities, cystic lesions, or calcifications. Ultrasound cannot accurately examine the deep structures of the knee and its accuracy depends on the radiologist's experience (operator-dependent). The reliability of ultrasound for the diagnosis of meniscal lesions varies considerably in the literature and existing results suggest that it is not satisfactory [1, 3–5]. It is therefore not a routine test for meniscal imaging. Only meniscal cysts are easily diagnosed and may be punctured and aspirated by ultrasound guidance. Rutten et al. [5] reported a sensitivity of 97%, a specificity of 86%, and accuracy of 94% in the study of meniscal cysts.

## 4. Arthrography and CT Arthrography

The reliability of arthrography of the knee for the diagnosis of meniscal lesions is well established (tears, bucket-handle tears, and meniscocapsular separation) with reliability between 83 and 94% [6, 7]. Arthrography was the reference technique in the 1970s and 1980s and was given up after 2000 to be replaced by MRI, which has the advantage of being noninvasive and without ionizing radiation. Nevertheless, today, arthrography can be associated with CT to perform CT arthrography, which provides complementary information to MRI. Indeed, thanks to continuous rotation scanning, spiral acquisitions provide high quality 2D multiplanar reconstructions with thin 0.5 mm slices. Coronal, sagittal, and even axial reconstructions can detect tears that are not visible on MRI, as well as meniscocapsular separations based on contrast enhancement between the meniscal wall and the peripheral capsule. This technique also provides a detailed analysis of tibiofemoral and patellofemoral joints with precise mapping of lesions. CT arthrography is not common in Anglo-Saxon countries but is still a gold standard technique for assessment of cartilage in meniscal lesions in Europe [8, 9]. This test has a sensitivity and specificity between 86% and 100% for the evaluation of meniscal lesions. CT arthrography of the knee is a safe technique that provides an accurate diagnosis of meniscal and cartilage injuries in patients who cannot undergo MRI (claustrophobia, pace-maker) or in the postoperative assessment of meniscal sutures and the condition of cartilage covering the articular surfaces [10].

## 5. Magnetic Resonance Imaging

Magnetic resonance imaging (MRI) is the most accurate and less invasive method for diagnosing meniscal lesions. It is more precise than a clinical examination and has influenced clinical practice and the treatment of patients by eliminating unnecessary diagnostic arthroscopies [11–13]. MRI has revolutionized imaging of the knee and become the “gold standard” for meniscal imaging. Its advantages are the analysis of meniscal lesions on all spatial planes, an excellent resolution using different sequences and high quality assessment of soft tissues. Its multiparametric characteristics allow visualization of specific injuries or structures depending on the sequences chosen. MRI allows characterization of meniscal lesions according to type, extension, association with a cyst, and meniscal extrusion as well as the evaluation of cartilage and subchondral bone. Thus, this technique provides a precise analysis of stability and the risk of propagation of the tear. It also determines whether the meniscal tear can be preoperatively repaired [13–16].

Overall, MRI has the following advantages:

- It does not expose the patient to ionizing radiation. Routine MRI does not require intravenous contrast agent administration, which may be associated with adverse events. No manipulation of joints is required. MRI is painless and can be performed in 20 minutes.
- Unlike CT arthrography, MRI does not require intra-articular administration of iodinated contrast agents. MRI has replaced arthrography and CT arthrography, except for patients who are too large to enter the MRI unit, or in patients with contraindications (intracranial aneurysm clips, metallic foreign bodies in the eye, or pacemakers and recent stents). MRI is also very useful for the diagnosis of residual meniscal lesions following meniscal surgery.

The disadvantages and contraindications are the following:

- MRI is limited in claustrophobic patients, obese patients (over 170 kg), or patients with pace-makers. With the use of open MRI machines and extremity MRI units, the number of patients who cannot undergo MRI due to claustrophobia or obesity has decreased. In patients with permanent contraindications to MRI, CT arthrography can be considered the alternative option.
- It is also limited by the presence of artifacts created by nearby orthopedic hardware. Resolution is hampered around the fixation by artifacts that depend upon the implant used. However, the use of nonferromagnetic metals such as titanium minimizes the artifacts in the postoperative knee [17]. Artifacts that hamper MRI results are considerably reduced if bioabsorbable screws are used. Moreover, artifacts associated with bioabsorbable screws tend to decrease over time. STIR sequences should replace fat-sat T2 sequence in the MRI protocol when imaging patients with orthopedic hardware.

### 5.1. MRI Technique

MRI machines with low, middle, and high field strengths (1, 1.5, or 3 T) can all provide accurate diagnostic images of meniscal lesions. For low-field strength MRI, the number of signals averaged must be increased to obtain a good quality meniscal image. However, this adjustment increases the imaging time and thus increases the risk of movement by the patient. Even the slightest amount of movement can degrade the images, which can compromise the ability to diagnose meniscal lesions [18–20].

Diagnostic sensitivity for medial meniscal lesions is between 86% and 96% with a specificity of 84% to 94%. The diagnostic sensitivity for the lateral meniscus is between 68% and 86%, with a specificity between 92 and 98% [16, 21–30]. The differences in sensitivity and specificity could be due to the sequences used, intraobserver variations or the size of the study population. Sensitivity for the detection of meniscal tears is usually higher for the medial meniscus, whatever the technique used [31]. Mackenzie et al. [32] reported that the overall sensitivity of MRI for meniscal lesions was 88% with a specificity of 94%. However, diagnostic errors are still possible on MRI. Using conventional coronal and sagittal spin-echo MR imaging, De Smet et al. [23] could not identify 6% of the meniscal tears, even in retrospect compared to arthroscopic findings. False-positive diagnoses due to healed tears or tears missed at arthroscopy occurred in 1.5% of menisci evaluated with MR imaging. Despite the large experience of radiologists, interpretation errors occurred in 21% menisci, due to misinterpretation of normal anatomic structures [23, 33, 34].

The most frequently used MRI is 1.5 tesla machine which provides high quality diagnostic images. There are very few results in the literature comparing musculoskeletal MRI with a 1.5 T and a 3.0 T machine. Faster image acquisition with the 3.0 T should result in more detailed images and improve the diagnostic accuracy [35, 36]. However, several studies [37–39] have shown that knee MRI with a 3.0 tesla machine is as sensitive and specific as with a 1.5 tesla machine. Moreover, 3T imaging has certain disadvantages including increased sensitivity to metallic artifacts.

### 5.2. Protocols and Imaging Views

The knee is generally extended in slight external rotation to facilitate imaging of the anterior cruciate ligament (ACL). High spatial resolution is necessary to show meniscal tears. This typically requires a field of view of 14 cm × 16 cm. For this review, we use 0.4 mm × 0.4 mm resolution for proton-density-weighted images and 0.5 mm × 0.5 mm resolution for fat sat T2-weighted images. An extremity coil optimizes the signal-to-noise ratio [13, 20, 40, 41].

Images should be obtained on all three planes: sagittal, coronal, and axial. Sagittal images are obtained with the knee in slight external rotation to visualize the anterior cruciate ligament (indeed, meniscal and ligament lesions are frequently associated). Several factors should be taken into account to optimize the imaging protocols. Although imaging on all three planes is useful, all sequences should not be performed on all planes. Usually T1-weighted sequences are performed on the sagittal plane while T2-weighted sequences are performed on all three spatial planes (sagittal, coronal, and axial) [40].

Sequences defining anatomical structures should be distinguished from those characterizing meniscal pathologies. Imaging of meniscal structures and contours is better with proton-density T2-weighted sequences:

- The so-called anatomical sequences: they are mainly T1-weighted proton density sequences. An MRI of the knee will nearly systematically include a sagittal T1-weighted image to evaluate the cruciate ligaments, the morphology of the menisci, the osteochondral structures, the extensor apparatus (patella, patellar tendon, and quadriceps), and the articular cavity.
- Sequences to identify pathologies: these sequences use fat suppression, either STIR or T-2 weighted fast spin-echo with specific fat suppression (T2 and T2 FSE Fat-Sat). This is the reference sequence for the analysis of intra-articular lesions: joint effusion, edematous infiltration, ligament or tendon tears, bone contusions, subchondral bone edemas, muscle lesions, and especially meniscal lesions.

There are other more specific sequences:

- T1-weighted Fat-Sat Gadolinium sequence (T1-weighted sequence with fat suppression and intravenous gadolinium administration). This sequence has the advantage of providing anatomical images while still being sensitive to all inflammatory and/or vascularized structures.
- T2-weighted sequence (gradient echo T2-weighted), this sequence is rarely used in other joints (shoulder and ankle) but is sometimes used in the knee. Its main value, besides good sensitivity for the diagnosis of meniscal tears [42], is mainly identifying signs of chronic bleeding in the form of hemosiderin deposition from villonodular synovitis.

The most reliable MRI sequences of the meniscus are proton density-weighted (FSE) sequences and T2-weighted and fast spin-echo T2-weighted sequences [43, 44] but also Rhô FSE Fat Sat sequences. T1-weighted sequences are less sensitive. Fast spin-echo is currently the imaging modality of choice.

### 5.3. Normal MRI of the Meniscus

**Figure 1:**
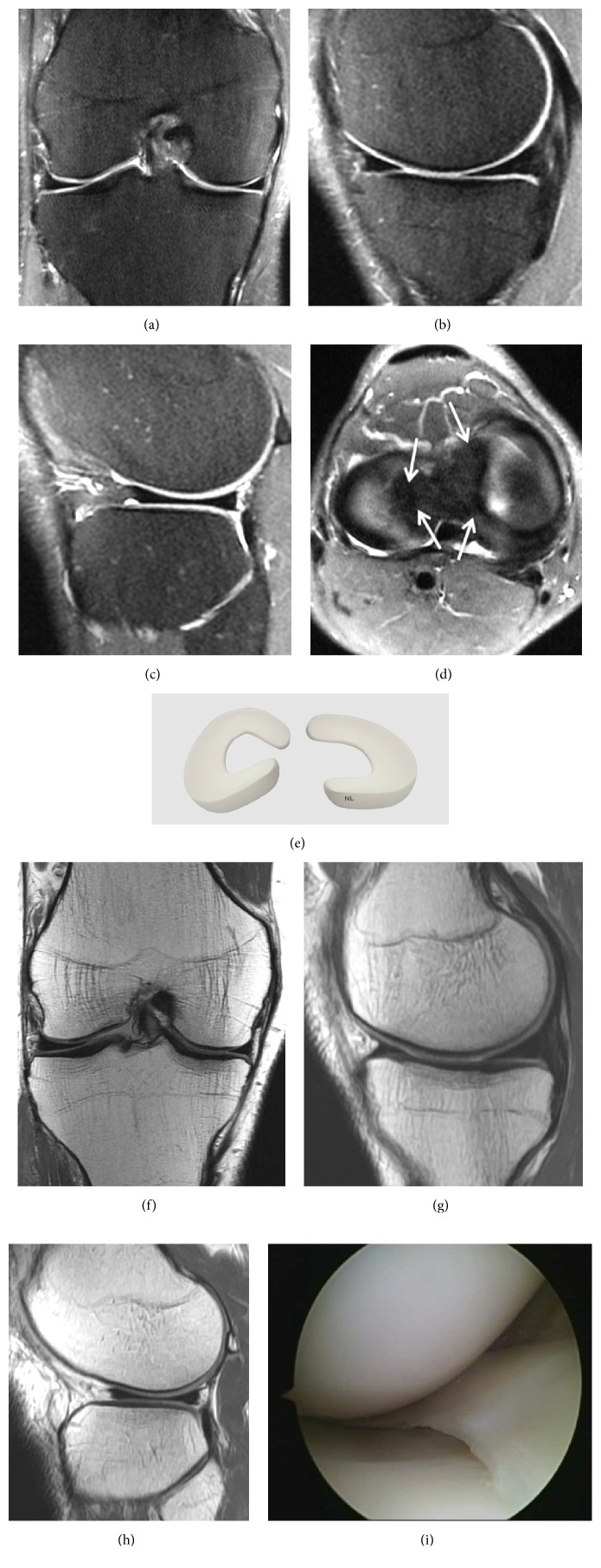
Normal meniscus on (a) Coronal T2 FSE Fat Sat MRI; (b) Sagittal T2 FSE Fat Sat MRI (the posterior horn is typically larger than the anterior horn medially); (c) Sagittal T2 FSE Fat Sat MRI (the horns of the lateral meniscus are equal in size and look like opposing triangles); (d) Axial T2 FSE Fat Sat MRI (the shapes of medial and lateral menisci differ, in attachment site (arrow). The medial meniscus is larger and has a more open C-shape configuration, with anterior and posterior tibial attachment sites separated by a greater distance compared to the lateral meniscus which has a more O-shape configuration); (e) three-dimensional diagram of the medial and lateral menisci; (f) coronal proton density weighted sequences; (g) sagittal proton density weighted sequences of the medial meniscus; (h) sagittal proton density weighted sequences of the lateral meniscus; (i) arthroscopic view of meniscus.

A normal meniscus is seen as a triangular shaped low intensity signal on classic T-1 and T-2 weighted sequences or on Fast Spin-Echo (FSE) sequences. The low intensity signal is due to a lack of mobile protons in the fibrocartilage of a normal meniscus (Figure 1).

In children, a grade 2 signal is often visualized in the posterior meniscal horns. This is considered to be normal and corresponds to the vascular system of the meniscus in children. This high intensity signal disappears in adulthood.

#### 5.3.1. Meniscal Stability

Anterior or posterior meniscofemoral ligaments (ligament of Humphrey and Wrisberg ligament) are present and visible on 33% of MRI images. Visualization of the ligament of Humphrey is better on sagittal images. It is sometimes observed on coronal images. The Wrisberg ligament is easier to see on posterior coronal images [5, 45]. The lateral meniscus is stabilized by the coronary ligament, the meniscofemoral ligament, the arcuate ligament, and the meniscotibial ligament [46]. Both menisci are also stabilized by the transverse ligament. If any of these supporting ligaments or the meniscus itself degenerates or is torn, the meniscus may become unstable. The meniscocapsular ligaments including the meniscofemoral and meniscotibial ligaments attach the menisci to the posterior femur and posterior tibial plateau, respectively [47].

### 5.4. Classification System of Meniscal Lesions

The features of meniscal degeneration are well codified on MRI. The use of Stoller and Crues 3-stage classification [45, 46] has been shown to be reliable, sensitivity: 87 to 97%, specificity: 89 to 98%, reliability: 88 to 95% [46, 48]. Only stage 3 degeneration (linear high intensity signal communicating with the joint) should be considered pathological. This MRI classification was developed in correlation with a histological model. The degenerative areas show a high intensity signal that varies depending on the location and severity of the meniscal lesion. This classification does not include peripheral capsular meniscal separations, which are not considered to be articular [45].

#### 5.4.1. Grade 1 Lesion

**Figure 2:**
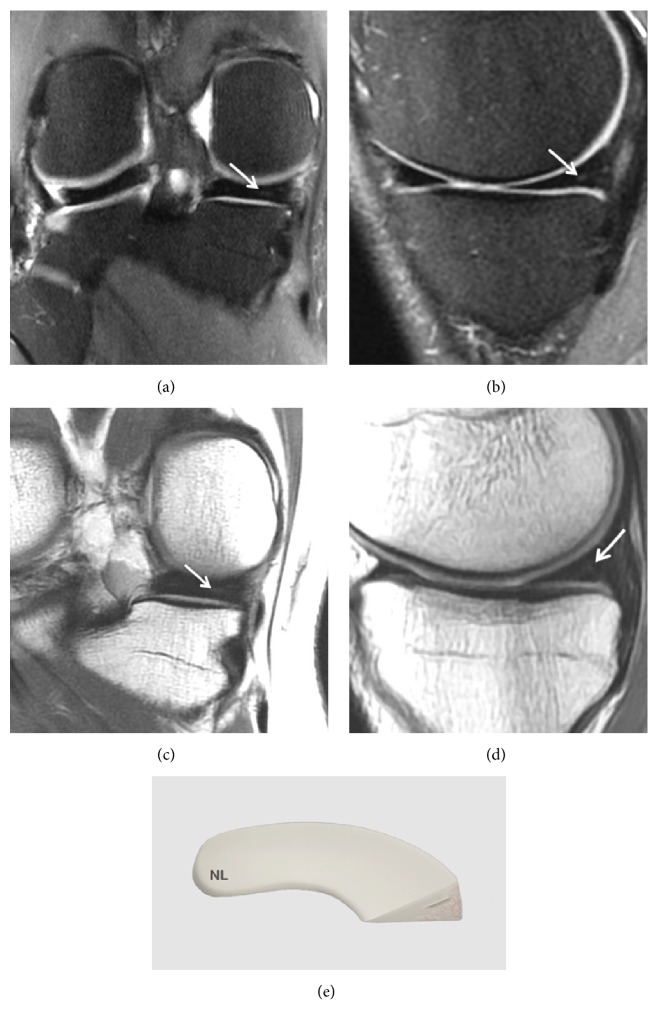
Grade 1 meniscal lesion (arrow) (a) Coronal T2 FSE Fat Sat MRI; (b) Sagittal T2 FSE Fat Sat MRI; (c) Coronal proton density weighted sequences; (d) sagittal proton density weighted sequences; (e) three-dimensional diagram.

A grade 1 lesion is a focal or diffuse nonarticular area (Figure 2).

This finding is correlated with early meniscal degeneration. The terms myxoid degeneration or hyaline degeneration are both used to describe these lesions.

#### 5.4.2. Grade 2 Lesion

**Figure 3:**
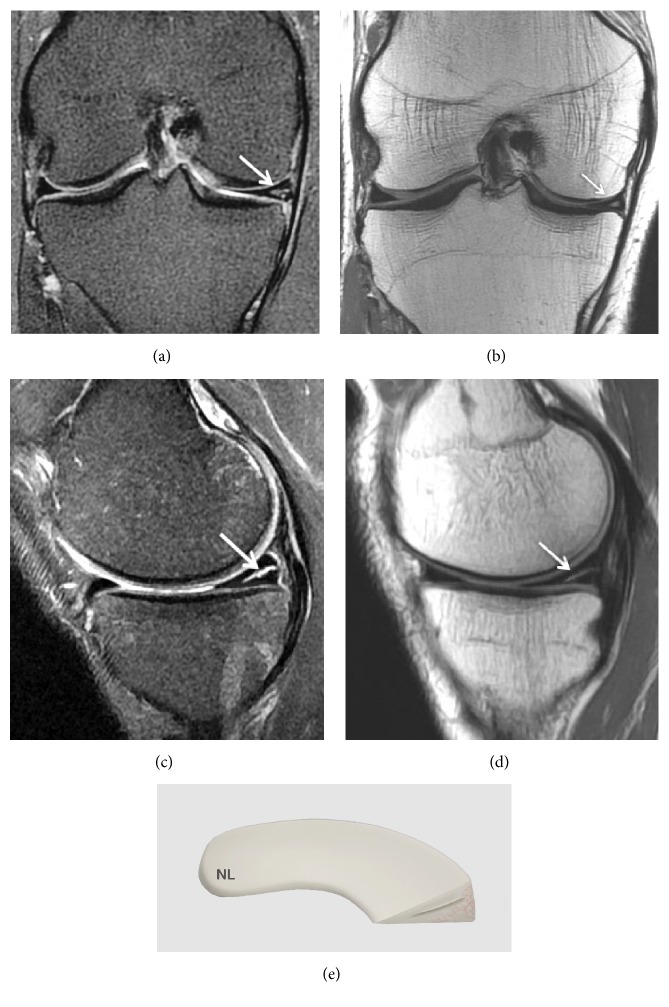
Grade 2 meniscal lesion on (a) Coronal T2 FSE Fat Sat MRI (arrow); (b) Coronal proton density weighted sequences (arrow); (c) Sagittal T2 FSE Fat Sat MRI (arrow); (d) sagittal proton density weighted sequences (arrow); (e) three-dimensional diagram.

A grade 2 lesion is a horizontal linear image in the body of the mensicus with a high intensity signal that extends to the inferior surface of the meniscus without involving it (Figure 3).

This abnormal signal is more extensive than in grade 1 degeneration but there is no cleavage or tear. Grade 2 degeneration is a progression of grade 1 degeneration. Patients are usually asymptomatic.

There are three types of grade 2 signals [45]:

 Type 2A is a linear signal without contact with an articular surface.
 Type 2B is an abnormal signal in contact with one of the articular surfaces on a single image.
 Type 2C is a very extensive signal without contact with an articular surface [46, 49].

#### 5.4.3. Grade 3 Tears

**Figure 4:**
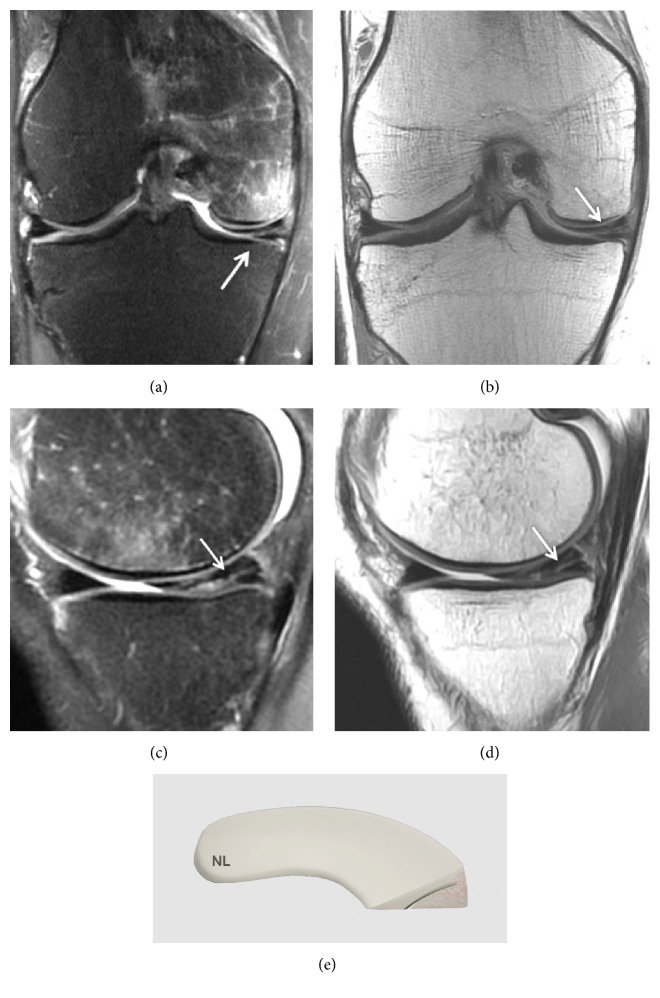
Grade 3 meniscal lesion on (a) Coronal T2 FSE Fat Sat MRI (arrow); (b) Coronal proton density weighted sequences (arrow); (c) Sagittal T2 FSE Fat Sat MRI (arrow); (d) sagittal proton density weighted sequences (arrow); (e) three-dimensional diagram.

Grade 3 corresponds to an abnormal signal in the meniscus that extends over a large part of the meniscus and communicates with at least one articular surface of the meniscus (Figure 4).

Nevertheless, approximately 5% of grade 3 lesions are intrameniscal with no real meniscal cleavage. They cannot be diagnosed on routine arthroscopy if surface extension is not identified preoperatively [50].

Besides this classification, there are two pathological criteria for meniscal tears. These two MRI criteria were established for the diagnosis of meniscal tears. If no prior surgery has been performed on the meniscus, the diagnostic accuracy of these criteria is more than 90% [13].

#### 5.4.4. Criteria 1

Criteria 1 correspond to an abnormal signal in the meniscus suggesting a tear that is found on at least two consecutive images. This corresponds to the “two-slice-touch rule” a concept with a positive predictive value of 94% for tears of the medial meniscus and 96% for the lateral meniscus. The positive predictive value was 55% and 36% for medial and lateral meniscal tears, respectively, when they are seen on a single image [4, 13, 51].

The abnormal signal intensity should be in contact with an articular surface, the superior or inferior or the tip (free end) of the meniscus. If the contact with the articular surface appears in two or more consecutive images, the diagnostic accuracy for a meniscal tear increases [13, 50].

#### 5.4.5. Criteria 2

Criteria 2 involve the morphology of the meniscus. A comprehensive understanding of the normal anatomy of the meniscus on MRI is necessary. Meniscal lesions are analyzed on the sagittal and coronal planes. Visualization of a meniscal tear on these two planes reduces the rate of false positives. However, several tears at the meniscocapsular junction may only be seen on one of these planes.

### 5.5. Description of Lesions: Size, Shapes, and Characteristics

Multiple images of meniscal tears should be translated into 3D images [13, 15]. Meniscal tears occur on two main planes: vertical and horizontal. The three basic shapes of meniscal tears are longitudinal, radial, and horizontal. Meniscal tears are either partial or full thickness (through all of the meniscal tissue).

#### 5.5.1. Vertical Tears

Vertical tears are perpendicular to the coronal plane of the meniscus and can be subdivided into peripheral longitudinal or radial tears. They usually occur following a trauma in young patients [52].

**Figure 5:**
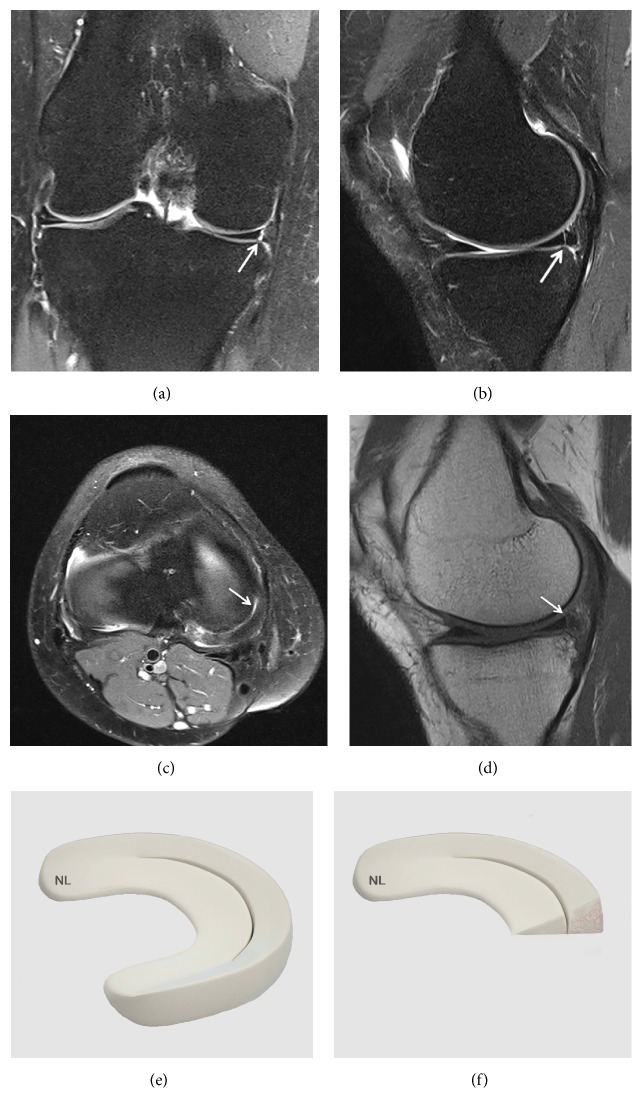
A vertical tear in the meniscal tissue communicating with the superior and inferior meniscal articular surfaces completely divides the meniscus into two parts. (a) Coronal T2 FSE Fat Sat MRI (arrow), (b) Sagittal T2 FSE Fat Sat MRI (arrow), (c) Axial T2 FSE Fat Sat MRI (arrow), and (d) sagittal T1-weighted sequence MRI. (e) Three-dimensional diagram shows a vertical and longitudinal tear of the meniscus. (f) Three-dimensional diagram shows a vertical tear.

A vertical tear in the meniscal tissue communicating with the superior and inferior meniscal articular surfaces completely divides the meniscus into two parts (Figure 5).

**Figure 6:**
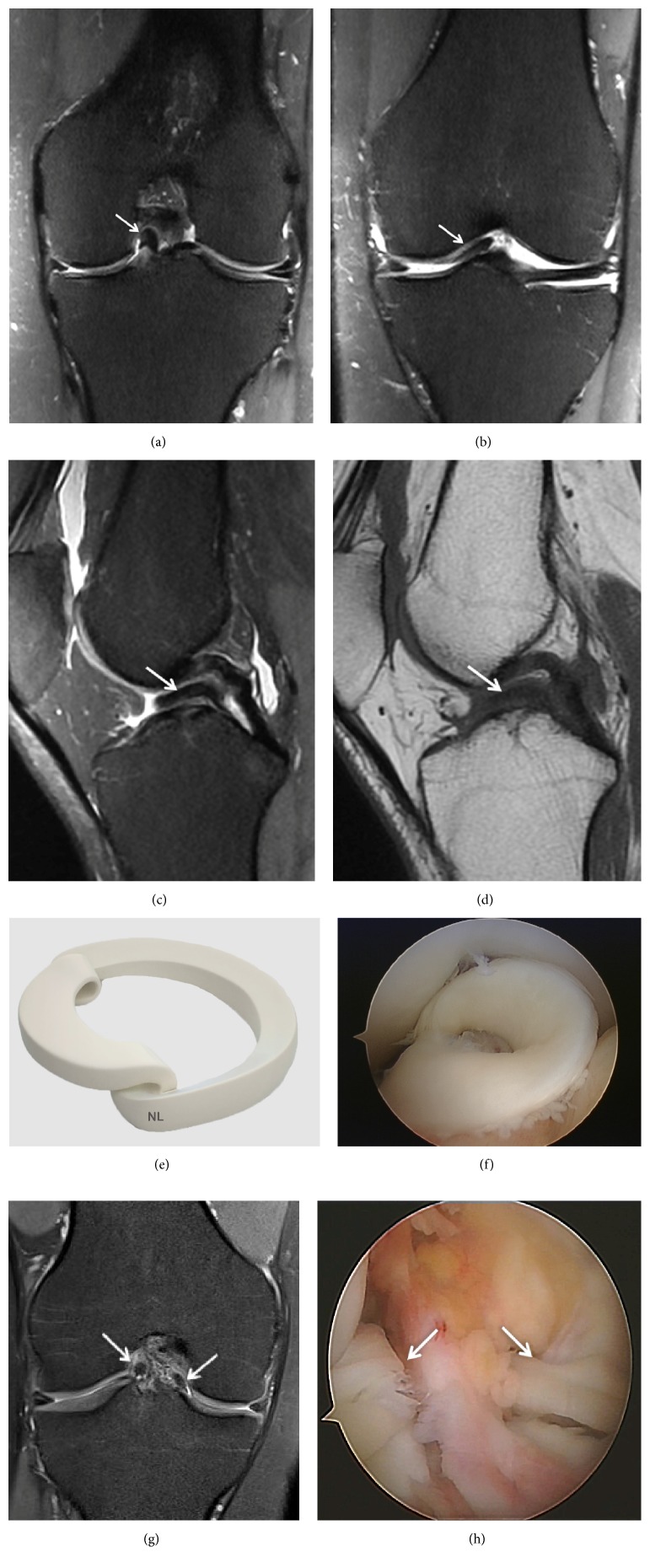
Displaced bucket-handle tear of the medial meniscus. (a) Coronal T2 FSE Fat Sat MRI shows a displaced bucket-handle fragment of the medial meniscus into the intercondylar notch of the knee (arrow). The remnant of the body of the meniscus is small; (b) Coronal T2 FSE Fat Sat MRI shows a displaced bucket-handle fragment of the anterior medial meniscus (arrow); (c) Sagittal T2 FSE Fat Sat MRI shows the “double PCL sign,” with a displaced fragment of a bucket-handle tear into the intercondylar notch of the knee; (d) Sagittal T1-weighted sequence MRI (arrow); (e) three-dimensional diagram shows a displaced bucket-handle tear of the medial meniscus; (f) arthroscopic view of a bucket-handle tear; (g) Coronal T2 FSE Fat Sat MRI shows a double displaced bucket-handle fragment of the medial and lateral meniscus into the intercondylar notch of the knee (arrow); (h) arthroscopic view of a double bucket-handle tear (arrow).

These tears can result in the development of bucket-handle tears (Figure 6) [20]. Vertical tears of the posterior horn may not be visible on sagittal images.

#### 5.5.2. Radial (or Transverse) Tears

**Figure 7:**
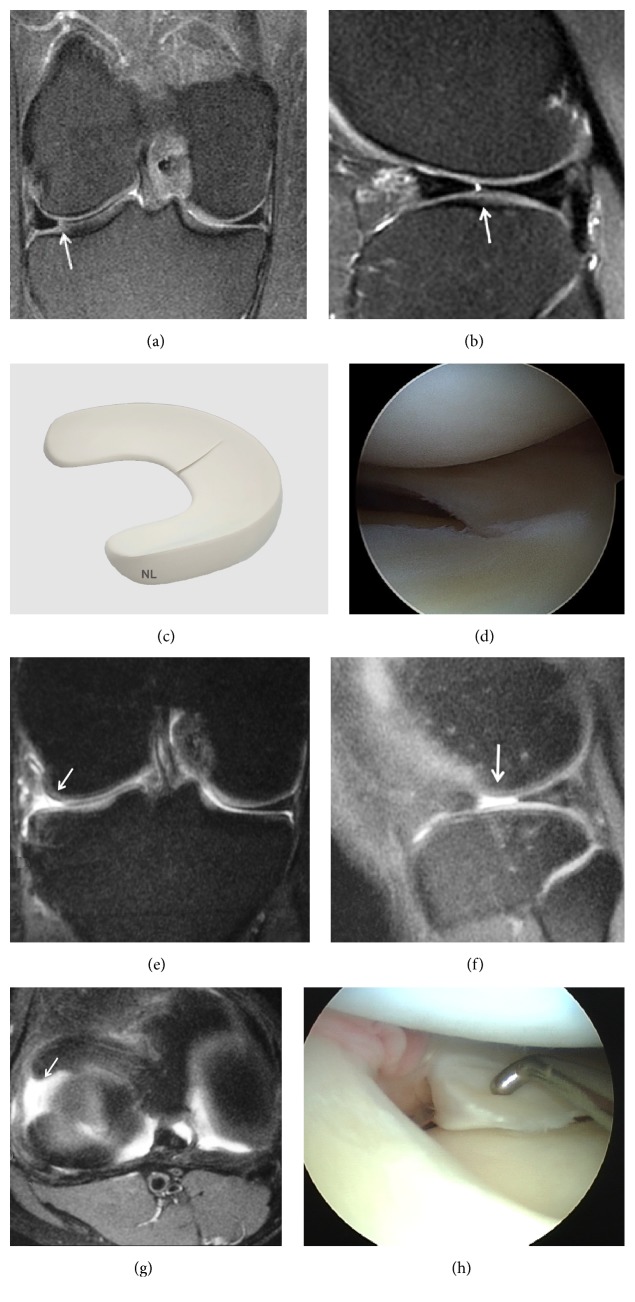
Radial tear involving the peripheral aspect of the meniscus. (a) Coronal T2 FSE Fat Sat MRI shows the vertical hyperintense signal (arrow) extends to both articular surfaces of the posterior horn of the medial meniscus; (b) Sagittal T2 FSE Fat Sat MRI shows the cleft sign of a radial tear; (c) three-dimensional diagram showing a radial tear involving the peripheral aspect of the meniscus; (d) arthroscopic view of the radial tear; (e) Coronal T2 FSE Fat Sat MRI: a part of the medial meniscus is not identified on the coronal image due to a large radial tear (arrow); (f) Sagittal T2 FSE Fat Sat MRI shows the large radial tear (arrow); (g) axial reconstruction showing the large radial tear (arrow) extending from the free edge into the posterior horn; (h) arthroscopic view of the large radial tear.

Radial tears are vertical tears that extend perpendicular to the main axis of the meniscus. The most frequent location is the middle segment of the meniscus (Figure 7).

This tear begins at the free edge of the meniscus and extends towards the periphery for a distance that varies [20]. A full thickness radial tear extends from the free edge towards the periphery of the meniscus (meniscal wall).

Small tears can be difficult to see on MRI. Radial tears represent a large percentage of the errors made in the interpretation of meniscal pathologies on MRI. The main feature of these tears is that they involve the free edge of the meniscal surface. Thus, if the inner point of the meniscal triangle is absent or blunted on one or more coronal images, a radial meniscal tear should be suspected. These tears are best seen on sagittal images.

**Figure 8:**
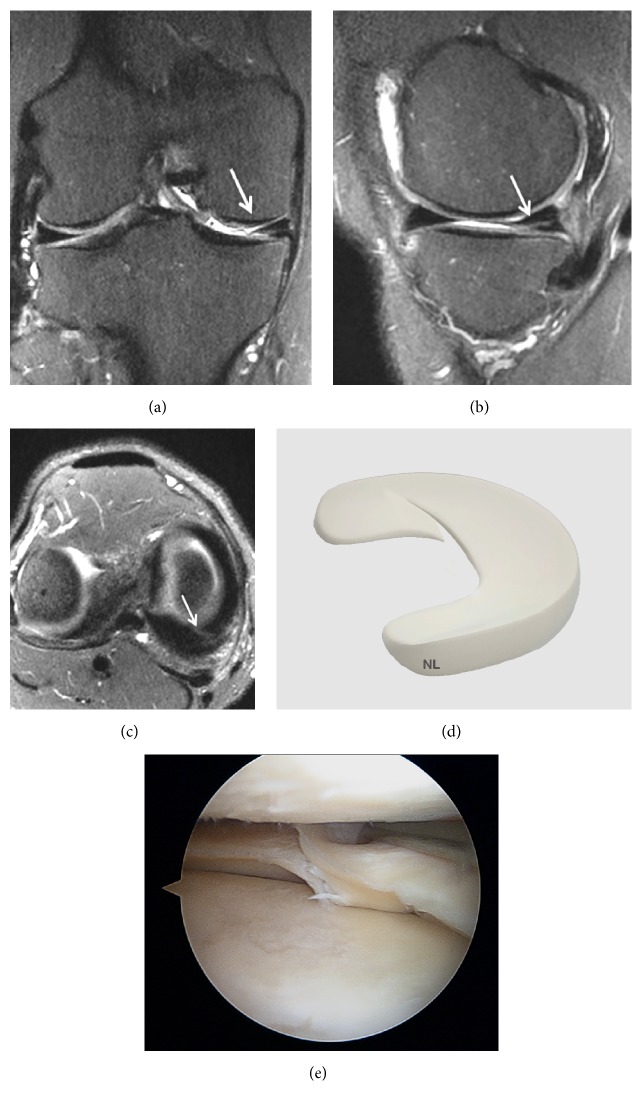
Oblique tears are a type of radial tear: (a) Coronal T2 FSE Fat Sat MRI: oblique tear of the body of the medial meniscus (arrow); (b) Coronal T2 FSE Fat Sat MRI: oblique-horizontal tear of the medial meniscus (arrow); (c) Axial T2 FSE Fat Sat MRI reconstruction showing the oblique tear of the posterior part of the medial meniscus (arrow); (d) three-dimensional diagram showing an oblique tear involving the peripheral aspect of the meniscus; (e) arthroscopic view showing a medial meniscus oblique tear.

Oblique tears are a type of radial tear (Figure 8).

**Figure 9:**
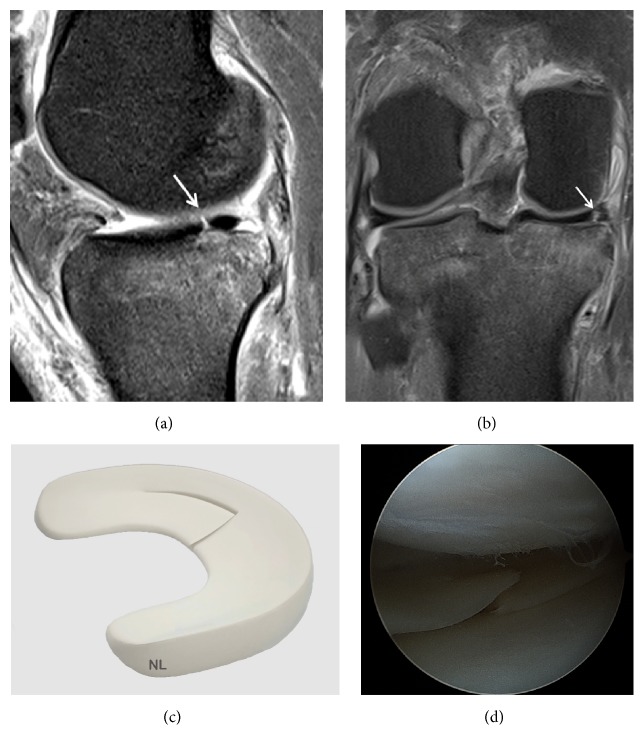
Radial tear extends towards the periphery to longitudinal meniscal tears; (a) Sagittal T2 FSE Fat Sat MRI (arrow); (b) Coronal T2 FSE Fat Sat MRI shows the longitudinal meniscal tears towards the periphery (arrow); (c) three-dimensional diagram showing the radial tear extends towards the periphery to longitudinal meniscal tears towards the periphery; (d) arthroscopic view showing a medial meniscus tear.

They begin on the free edge of the meniscus then continue longitudinally (Figure 9), similar to longitudinal meniscal tears, and the tear extends towards the periphery.

These oblique tears are the most frequent meniscal tear [13, 15, 46]. Oblique radial tears of the posterior horn of the lateral meniscus are often associated with ACL tears [53].

#### 5.5.3. Horizontal Tears

**Figure 10:**
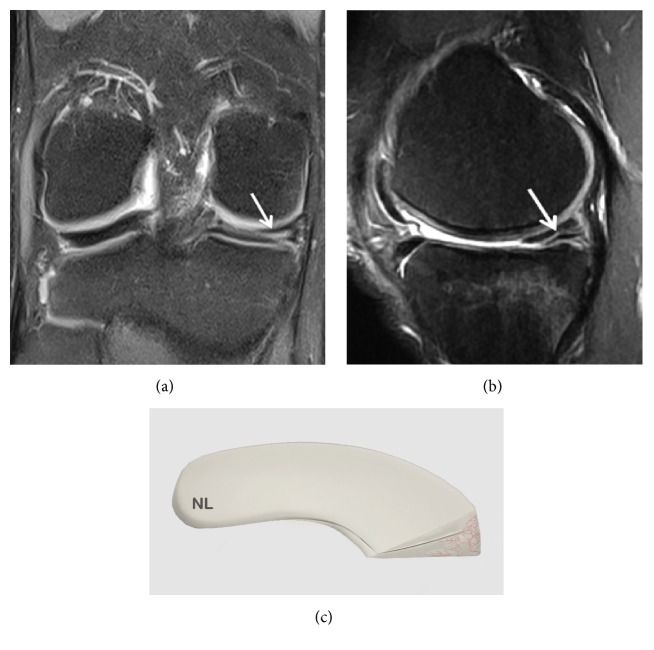
Horizontal tears are also called cleavage or fish-mouth tears. (a) Coronal T2 FSE MRI: horizontal tear (arrow) of the body of the medial meniscus; (b) Sagittal T2 FSE MRI (arrow); (c) three-dimensional diagram.

Horizontal tears are also called cleavage or fish-mouth tears (Figure 10).

They divide the meniscus into two superior and inferior parts. They usually begin on the underside of the meniscus [20]. Although horizontal tears may appear to extend deep into the meniscus on MRI, they may only be several millimeters deep on arthroscopy. When the tear extends to the periphery of the meniscus, to the meniscosynovial border, this can form a meniscal cyst. Most of these tears are degenerative and occur in elderly patients with osteoarthritis.

#### 5.5.4. Complex Tears

Complex tears are a combination of longitudinal, radial, and horizontal tears. Several tears may be present simultaneously in the meniscus, involving different parts of the same region or several regions. One common complex tear includes a horizontal and radial tear. It is nearly always degenerative [13, 15].

#### 5.5.5. Meniscus Posterior Horn Avulsion

**Figure 11:**
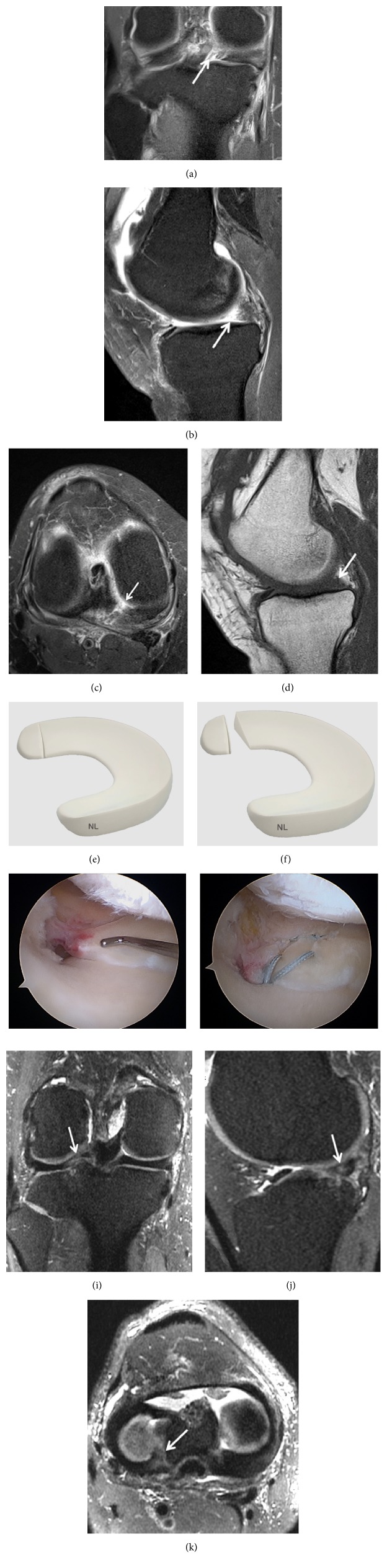
Meniscus posterior horn avulsion. (a) T2-weighted fat-saturated images showing a complete posterior root tear of the medial meniscus (arrow); (b) Ghost meniscus sign. The posterior horn of the medial meniscus has been replaced with triangular high signal intensity on the T2-weighted fat-saturated sequence (arrow); (c) axial reconstruction showing the large posterior horn avulsion (arrow) with high signal intensity on the T2-weighted fat-saturated sequence; (d) the posterior horn of the medial meniscus is not identified on the sagittal T1 (arrow); (g) arthroscopic view showing a displaced medial meniscus root tear; (h) arthroscopic view showing a suture of the medial meniscus root tear; (i) identification of root tears of the lateral meniscus can be more difficult on the coronal T2-weighted fat-saturated sequence; (j) ghost meniscus sign is less significant on the sagittal T2-weighted fat-saturated sequence (arrow); (k) axial reconstruction showing the posterior horn avulsion (arrow).

It is not always easy to diagnose a meniscus avulsion on MRI (Figure 11).

The diagnostic sensitivity of MRI for the detection of root avulsion of the posterior horn is only 66.3%, which is not specific enough to determine the type of tear [54]. However, recent studies have helped improve the sensitivity and specificity of these tears [47, 55–59]. Lee et al. [59] have proposed a diagnostic assessment based on three MRI signs: a “ghost meniscus” on the sagittal plane (100% detection rate), the “vertical linear defect” (truncated aspect) on the coronal plane (100%), and the “radial linear defect” on the axial plane (94%).

#### 5.5.6. Displaced Meniscal Fragments

Fragments or displaced meniscal loose bodies occur in 9–24% of meniscal tears. All forms of tears can result in displaced fragments [60]. Diagnosis by MRI is based on visualization of the tear with a missing portion of the meniscus and of the displaced meniscal fragment [61].

**Figure 12:**
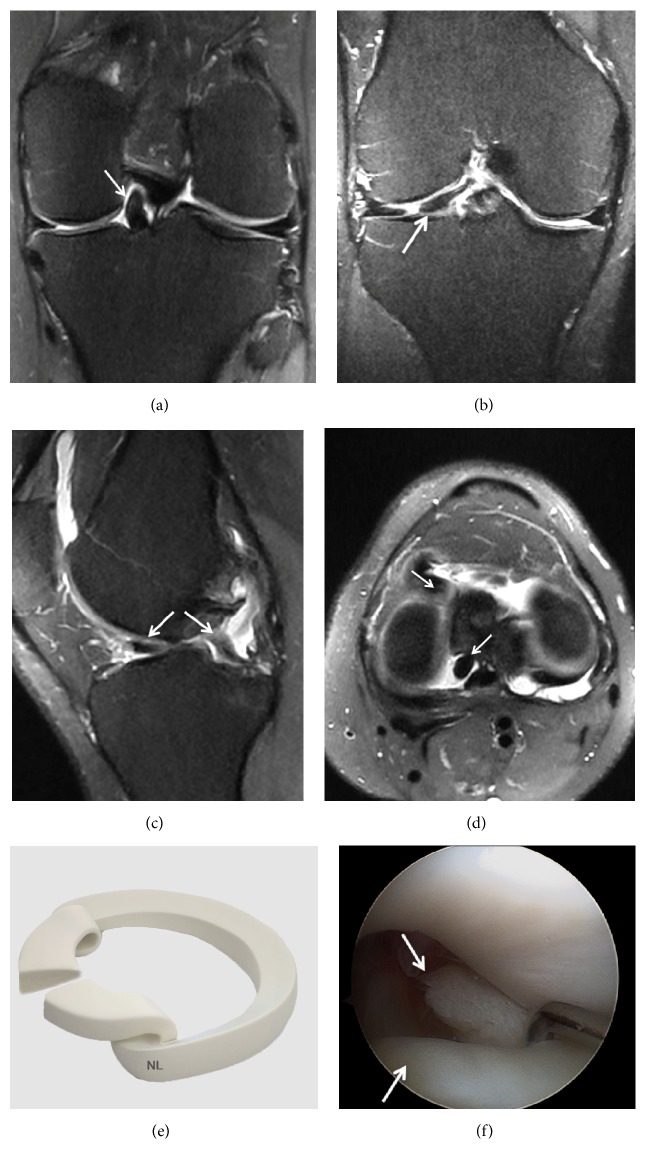
Displaced bucket-handle tear of the medial meniscus with tear from the middle part of the meniscus. (a) Coronal T2 FSE Fat Sat MRI: large meniscal fragment (arrow) seen within the intercondylar notch; (b) Coronal T2 FSE Fat Sat MRI: flap tears displaced horizontal under surface tear of the body and anterior horn of the medial meniscus with a flipped fragment (arrow); (c) Sagittal T2 FSE Fat Sat MRI showing a complex tear with a displaced fragment (arrow); (d) Axial T2 FSE Fat Sat MRI reconstruction showing the 2 flap tears of the displaced bucket-handle; (e) three-dimensional diagram; (f) arthroscopic view showing the rupture and the displaced bucket-handle tear.

*(i) Bucket-Handle Tear*. The bucket-handle tear is caused by a full thickness vertical-longitudinal tear. The fragment (which may or may not be displaced) separated by the meniscal wall, on the axial images, looks like a bucket handle. These tears account for 10% of all meniscal tears [40, 61–63]. The diagnostic accuracy of MRI for bucket-handle tears (Figure 6) is good and the displaced fragment can be clearly visualized in the intercondylar notch on coronal but also on sagittal images when searching for the double posterior cruciate ligament sign (double PCL sign) [50, 62, 64]. These tears can be classified as simple vertical longitudinal tears, displaced or not, torn or not from the middle part of the meniscus (Figure 12) and sometimes with double or triple bucket handles.

These tears are three times more frequent in the medial meniscus than in the lateral meniscus and may be associated with ACL tears.

Pseudohypertrophy of the anterior horn of the lateral meniscus occurs when the anterior horn of the meniscus seems abnormally large. The posterior horn of the lateral meniscus is abnormally weak. This indicates that part of the meniscus has tipped forward into a bucket-handle tear.

**Figure 13:**
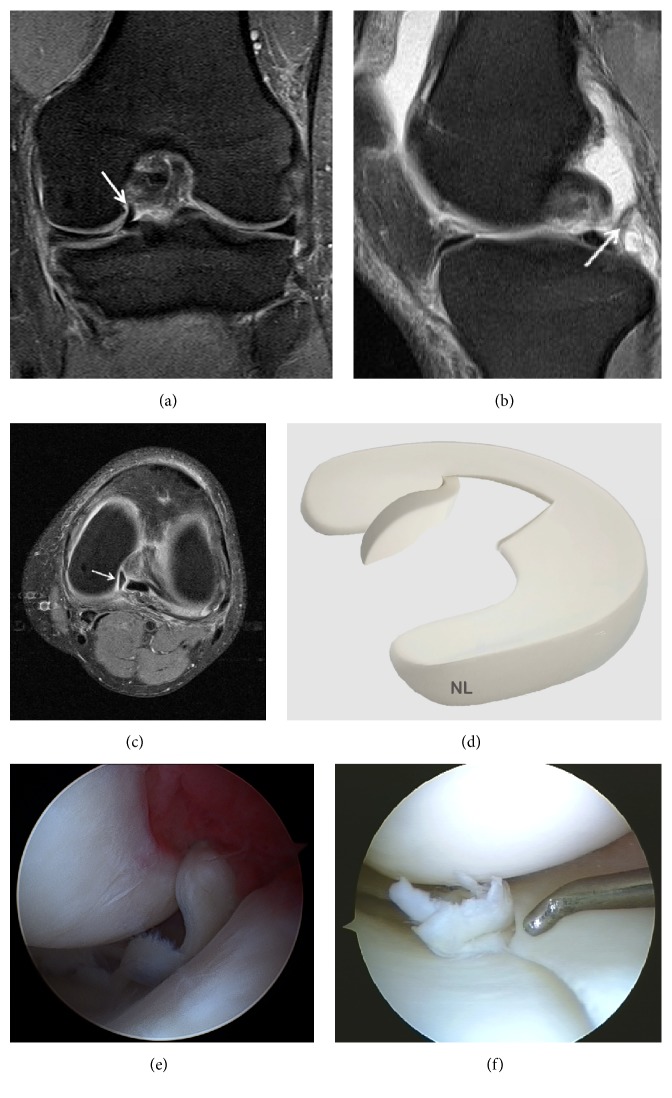
Meniscal fragments from horizontal meniscal tears can sometimes be displaced in relation to the body of the meniscus, slipping above or below the rest of the meniscal surface. (a) Coronal T2 FSE Fat Sat MRI showing a displaced fragment of the medial meniscus; (b) a meniscal fragment (arrow) is seen posterior to the PCL in Sagittal T2 FSE Fat Sat MRI; (c) axial reconstruction showing meniscal fragment (arrow) on the T2-weighted fat-saturated sequence; (d) three-dimensional diagram showing the meniscal fragments; (e) arthroscopic views of a displaced tear of the medial meniscus in the intercondylar notch; (f) arthroscopic views of a displaced tear of the medial meniscus in the underlying posteromedial tibial plateau.

*(ii) Meniscal Fragments*. Meniscal fragments from horizontal meniscal tears can sometimes be displaced in relation to the body of the meniscus, slipping above or below the rest of the meniscal surface (Figure 13).

**Figure 14:**
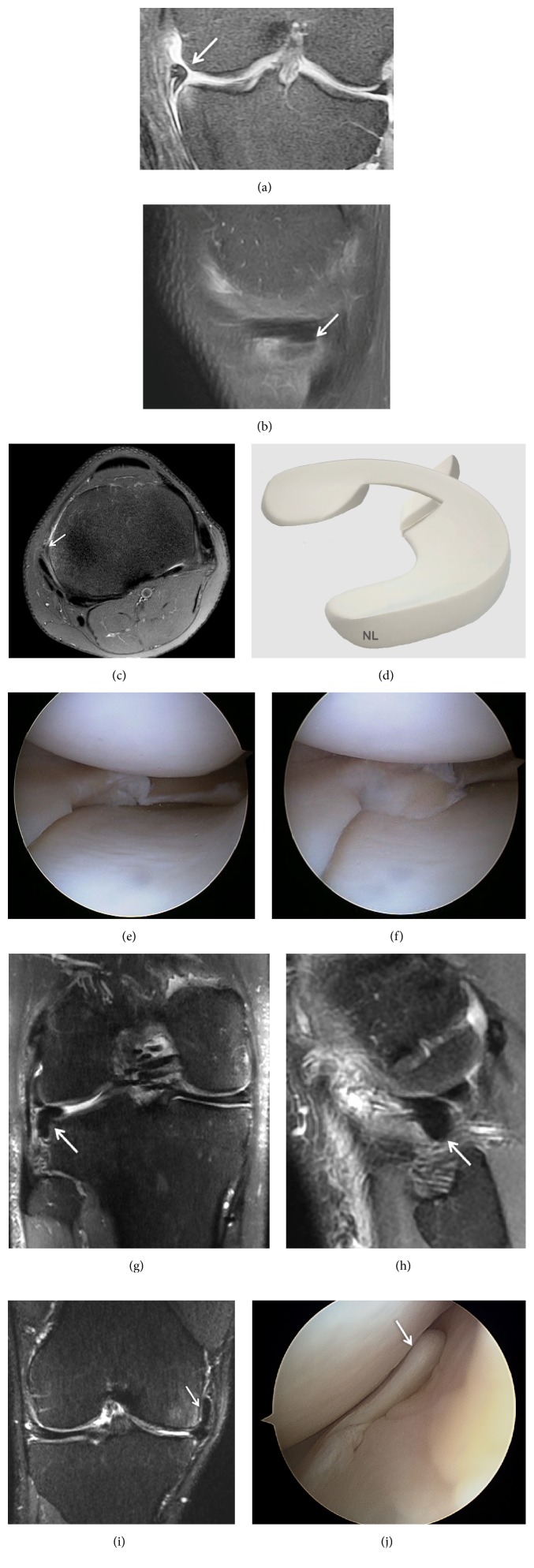
Meniscal fragments from horizontal meniscal tears displaced under the medial or lateral meniscus. The displaced fragment blocks the peripheral edge of the tibial plateau and the deep part of the MCL or LCL. (a) Coronal T2 FSE Fat Sat MRI showing a displaced horizontal undersurface tear of the body of the medial meniscus with a flipped fragment (arrow) along the undersurface of the native meniscus and extending under MCL; (b) Sagittal T2 FSE Fat Sat MRI showing a displaced fragment of the medial meniscus (arrow); (c) axial reconstruction showing the flipped fragment (arrow) under MCL on the T2-weighted fat-saturated sequence; (d) three-dimensional diagram showing a displaced tear of the medial meniscus; (e) arthroscopic views of a displaced tear of the medial meniscus under the meniscus; (f) arthroscopic view of the medial meniscus tear under the meniscus reduced in intra-articular lesion; (g) Coronal T2 FSE Fat Sat MRI showing a displaced fragment of the lateral meniscus (arrow); (h) Sagittal T2 FSE Fat Sat MRI showing a large fragment of the lateral meniscus under the LCL; (i) complex tear with a displaced fragment (arrow) coursing into the superior recess in Coronal T2 FSE Fat Sat MRI; (j) arthroscopic views of a displaced tear of the medial meniscus into the superior recess.

These fragments generally concern the medial meniscus [60]. Inferomedial displaced fragments under the medial meniscus are rare. When the displaced fragment blocks the peripheral edge of the tibial plateau and the deep part of the medial cruciate ligament, it cannot be seen on arthroscopy because the surface of the meniscus appears to be intact. On the other hand, it is more often visible on coronal images (Figure 14).

Superior or inferior displacement of a small meniscal fragment from a vertical tear is less frequent.

#### 5.5.7. Meniscocapsular Separation

Meniscocapsular separation is a tear of the periphery of the meniscus at the meniscosynoval junction. This usually involves the capsular attachment of the posterior horn of the medial meniscus. MRI is much less reliable than arthroscopy for the diagnosis of meniscocapsular separation, positive predictive value: PPV of 9% for the medial meniscus and 13% for the lateral meniscus [65].

Meniscocapsular separation is frequently associated with knee ligament tears. These entities can heal spontaneously because of the rich vascularization on the periphery of the meniscus, depending on the site of separation in relation to the area of vascularization of the connective tissue.

#### 5.5.8. Meniscal Cyst

**Figure 15:**
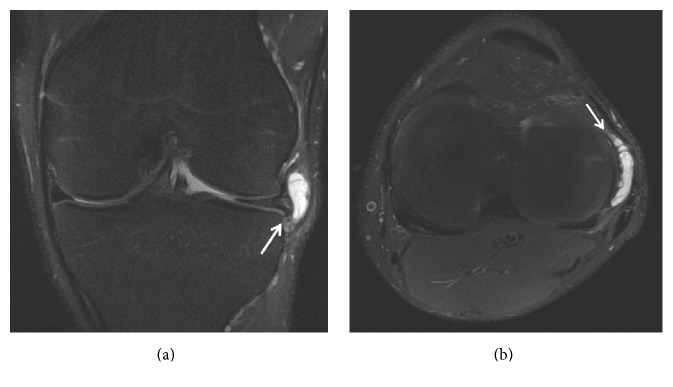
Lateral meniscal cysts: (a) lateral meniscal cysts are usually located at the anterior meniscal horn (coronal T2 FSE MRI sequences); (b) Axial T2 FSE Fat Sat MRI reconstruction showing the lateral meniscal cysts (arrow).

Meniscal cysts occur more frequently in the medial compartment [66]. Symptoms of medial parameniscal cysts are more frequent because of their location near the medial collateral ligament. The incidence is between 2% and 8%, and these cysts are usually found in men between 20 and 40 years old. Medial meniscal cysts are usually found at the posterior horn [20], while lateral meniscal cysts are usually located at the anterior meniscal horn (Figure 15).

Tears are usually horizontal and extend to the periphery of the meniscus, allowing synovial fluid to leak from the joint into the parameniscal tissue and form a meniscal cyst. Sometimes the cyst can be limited to the meniscus. This is called an intrameniscal cyst.

The parameniscal cyst located adjacent to the lateral anterior meniscal horn is less at risk of an underlying meniscal tear than cysts in other meniscal locations [67]. It is important to recognize the link between meniscal cysts and tears. If the cyst is treated without treating the tear, the cyst can recur.

#### 5.5.9. Meniscal Extrusion

Meniscal extrusion of the tibiofemoral joint space has been reported in elderly patients with clinically symptomatic osteoarthritis of the knee. In this group, meniscal extrusion preceded the degenerative joint disease [68]. Following meniscal extrusion, direct impaction of the tibial and femoral cartilages increases progression to osteoarthritis.

Tibiofemoral cartilage damage and leg malalignment increase the risk of meniscal extrusion. Poor alignment increases the loads on the meniscal surface that can lead to extrusion. Varus and valgus malalignment are associated with medial and lateral meniscal extrusion, respectively [68].

#### 5.5.10. Postoperative Meniscus and MRI

The diagnosis of a recurrent tear is more complex in patients who have undergone partial meniscectomy or meniscal repair and coronal and sagittal T2-W FSE Fat-Sat sequences are recommended. In sutured menisci, a persistent linear hypersignal in the suture zone makes it difficult to obtain the differential diagnosis between a recurrent tear and a separation that is in the process of healing [69]. If there was more than 25% resection of the meniscal surface or meniscal repair, most authors advise using MR arthrography [12]. Following meniscectomy, the remaining meniscus can present with a heterogeneous signal and irregular borders without being pathological. The diagnostic accuracy of MR-arthrography for recurrent meniscal tears is 88%, while it is 66% with routine MRI. During extensive meniscectomies, MR-arthrography is more accurate than simple MRI. Magee [70] has showed recently that the combined use of MR and MR arthrogram imaging was 98% sensitive and 75% specific in the diagnosis of a meniscal retear.

#### 5.5.11. Discoïd Meniscus

**Figure 16:**
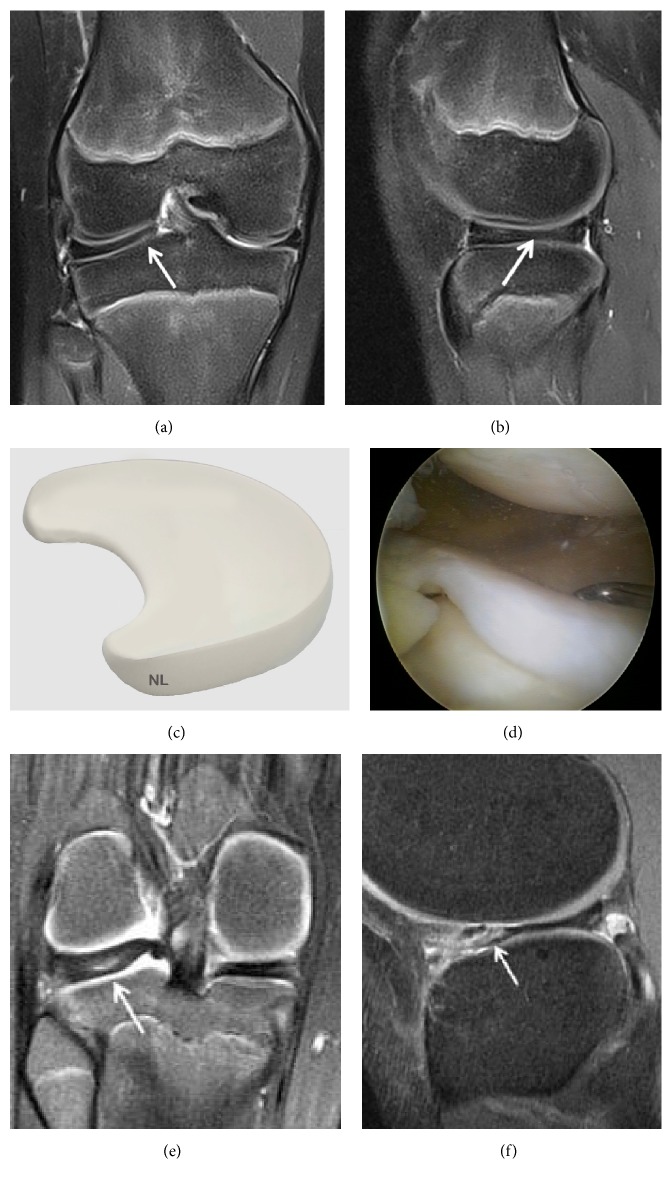
Discoid lateral meniscus. (a) Coronal T2 FSE Fat Sat MRI showing meniscal enlargement. The lateral meniscal body (arrow) is enlarged and has a more slab-like configuration compared to the normal-appearing triangular medial meniscal body; (b) Sagittal T2 FSE Fat Sat image of the lateral meniscus demonstrating persistence of the bow tie appearance on the more central slices rather than converting into 2 opposing triangles; (c) three-dimensional diagram showing a discoid lateral meniscus; (d) arthroscopic views of a discoid lateral meniscus; (e) posterior cystic degeneration in a discoid lateral meniscus; (f) anterior cystic degeneration in a discoid lateral meniscus.

Differentiation between true discoid meniscus and a meniscus that is a little larger than normal can be difficult (Figure 16).

The three types of discoid meniscus are classified as complete, incomplete, and Wrisberg discoid meniscus. The amount of tibial plateau coverage varies between complete and incomplete discoid meniscus. The Wrisberg variant is the least frequent anomaly (it lacks the normal posterior coronary ligament and capsular attachments). This ligament is mobile and can sublux [71].

On sagittal images, a discoid meniscus has a thickened bowtie appearance on three consecutive sagittal images. The anterior horns and the normal meniscus are visible on several MRI images near the intercondylar notch. With a complete discoid meniscus this difference is not seen. The normal meniscus rapidly narrows from the outer periphery to the center. The presence of an equally or nearly equally high meniscus on 2 adjacent 5 mm thick images is a sign of discoid meniscus [45].

Coronal MRI images are more sensitive for the diagnosis of discoid meniscus by showing meniscal enlargement. An asymmetric discoid meniscus can have an enlarged meniscal body on coronal images but have normal posterior and anterior horns on sagittal images, emphasizing the necessity of coronal images. Discoid meniscus is accurately diagnosed on MRI (PPV 92%) [72].

## 6. 3D Isotropic Turbo Spin-Echo MRI

3D isotropic Turbo Spin-Echo MRI was developed to create multiplanar reformatted images to obtain reconstructions of other spatial planes from a single plane acquisition or even on the axis of a structure defined as a ligament. Besides visualizing 2D and 3D structures, this technique also reduces overall MRI examination time [8, 73–76]. Moreover, small anatomical structures can be visualized with 3D MRI and the partial volume effect can be minimized by thin slices. Finally, the field of study can cover the entire area of interest without interslice gaps [8, 77, 78]. Thus, 3D MRI has received increasing attention for musculoskeletal imaging because most anatomical structures are small and facing in different directions, often oblique, especially the ACL [78, 79]. Until recently, 3D isotropic images were based on gradient-echo imaging which has certain disadvantages, such as increasing the risk of artifacts and a lack of contrast between normal and diseased tissue [80, 81]. Recently, turbo-spin-echo (TSE) sequences have been shown to provide 3D isotropic images in acceptable acquisition times [82–84]. The knee is one of the most frequent applications of 3D isotropic sequences. TSE is considered to be the best sequence to evaluate the internal structures of the knee because of its high definition of tissue contrast [55, 85]. The diagnostic accuracy of TSE sequences is comparable to routine spin-echo MRI, for cartilage coverage, menisci, ligaments and subchondral bone [86–88]. Recent studies have shown that standardized TSE sequences make it possible to detect more meniscal lesions and in particular the first stages of osteoarthritis [89, 90].

## 7. Conclusion

MRI is the most accurate and least invasive tool for the diagnosis of meniscal tears. This knee imaging technique is the “gold standard” for the analysis of meniscal lesions. It allows confirming and characterizing the meniscal lesion. The diagnostic arthroscopy alone therefore has no place in the analysis of meniscal lesions of the knee. However, the therapeutic arthroscopy is a feasible treatment of meniscal lesions of the knee. The perfect knowledge of different meniscal lesions described in this paper allows the clinician to adapt treatment, medical or surgical, specifically for each lesion. New 3D MRI in three dimensions with isotropic resolution should help improve the diagnosis of meniscal tears.
